# Transcriptional data: a new gateway to drug repositioning?

**DOI:** 10.1016/j.drudis.2012.07.014

**Published:** 2013-04

**Authors:** Francesco Iorio, Timothy Rittman, Hong Ge, Michael Menden, Julio Saez-Rodriguez

**Affiliations:** 1 EMBL – European Bioinformatics Institute, Wellcome Trust Genome Campus, Cambridge CB10 1SD, UK; 2 Dept of Clinical Neurosciences, Herchel Smith Building, Forvie Site, Addenbrooke's Hospital, Robinson Way, Cambridge CB2 0SZ, UK; 3 Dept of Applied Mathematics and Theoretical Physics, Centre for Mathematical Sciences, Wilberforce Road, Cambridge CB3 0WA, UK; 4 Cancer Genome Project, Wellcome Trust Sanger Institute, Wellcome Trust Genome Campus, Cambridge CB10 1SD, UK

## Abstract

Recent advances in computational biology suggest that any perturbation to the transcriptional programme of the cell can be summarised by a proper ‘signature’: a set of genes combined with a pattern of expression. Therefore, it should be possible to generate proxies of clinicopathological phenotypes and drug effects through signatures acquired via DNA microarray technology.

Gene expression signatures have recently been assembled and compared through genome-wide metrics, unveiling unexpected drug–disease and drug–drug ‘connections’ by matching corresponding signatures. Consequently, novel applications for existing drugs have been predicted and experimentally validated.

Here, we describe related methods, case studies and resources while discussing challenges and benefits of exploiting existing repositories of microarray data that could serve as a search space for systematic drug repositioning.

## Highlights

► Can every biological state be represented by a given gene expression signature? ► Recently, signatures have been used as proxies of clinicopathological phenotypes. ► Drug–drug/drug–disease ‘connections’ have been inferred by signature matching. ► This allowed the prediction of new application for already approved drugs. *►* A massive amount of public transcriptional data is ready to be exploited in this way.

## Introduction

During past decades the main strategy of drug development has been high-throughput screening of different molecules to identify lead compounds showing activity against single therapeutic targets and pathways. However, the ratio of successfully identified drugs to screened molecules has decreased dramatically over the years [1]. Furthermore, targeting individual elements of pathogenic pathways is not always a successful approach for tackling the complexities of the disease state; even when a target pathway is identified, a suitable drug might not be found. For example, in Alzheimer's disease the ‘amyloid hypothesis’ has driven the search for drugs that stop aggregation of pathogenic beta-amyloid, which generates potentially toxic oligomers and plaques, but so far these efforts have not led to a successful disease-modifying treatment [2]. In addition, the cost of bringing an effective drug to the market is large and growing with a significant portion of investment needed in the research and development phase [3]. Many promising molecules never come into clinical use because they show unfavourable pharmacokinetic properties or cause adverse reactions in humans. As a consequence there is a pressing need to identify successful treatments for many diseases in innovative ways that could overcome these drawbacks.

Drug repositioning [4] is a potential alternative to new drug discovery that promises to address some of these issues by identifying new therapeutic applications for existing drugs. One of the advantages of reconsidering established drugs is that they have already been approved and, hence, they can potentially be re-marketed in a faster and more cost-efficient way – by skipping Phase I clinical trials [5]. Moreover, pharma company pipelines already include many drug candidates that have passed Phase I trials but were not successful in Phase II or III (i.e. being safe but not sufficiently effective in treating the condition they were originally designed for). This implies that the search basin for repositionable drugs is vast and much larger than the set of approved drugs [6].

Most cases of successfully repositioned drugs can be linked to serendipity, such as the classic example of sildenafil which is used to treat erectile dysfunction but was originally developed as a cardiovascular drug [7]. However, systematic approaches have recently been proposed. Most of these are based on the principle that shared properties between compounds could hint at similar efficacy or commonality in their mode of action (MoA). Successful strategies based on this assumption have been devised and published in different areas of computational drug discovery: from chemoinformatics [8] and structural bioinformatics [9] to text mining and meta-data analyses [10] and, recently, genome-wide association studies [11]. Many of these strategies benefit from recent advances in data integration and systems biology [12] and among them a new trend has emerged over the past few years that is based solely on the analysis of gene expression data [13].

The traditional ‘central dogma’ of molecular biology is the principle of genes encoding mRNA that is translated into proteins. This defines a biological information flow that, moving through levels of increasing complexity and emerging properties, links the underlying genetic make-up of the cell to its clinicopathological state [13]. In such a context, transcriptional profiling enables the capture of a multidimensional view of this complexity at an intermediate level, reflecting genomic and environmental effects.

**Figure 1:**
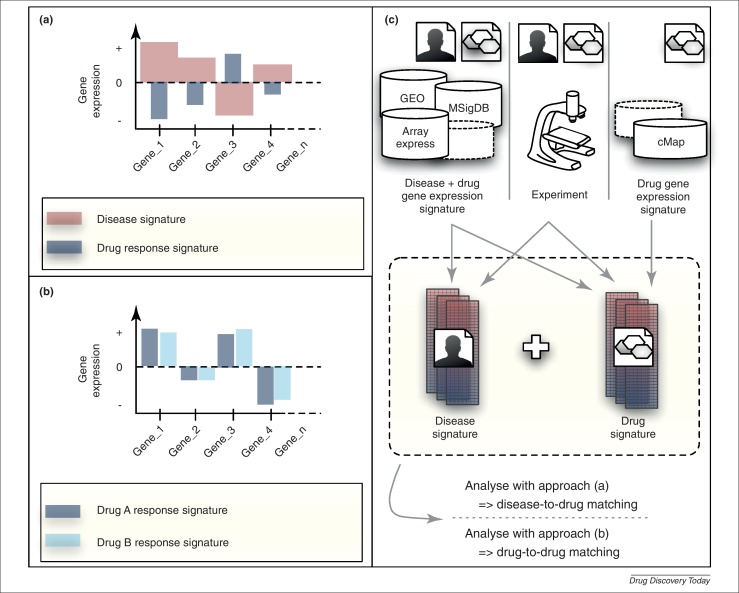
Signature reversion **(a)** and guilt-by-association **(b)** approaches in gene-expression-based drug repositioning. In (a) the aim is to identify a drug where the effect on transcription is opposite to a disease signature. In (b) drugs eliciting similar gene expression signatures are sought and hypothesised to share a common mode of action. Many publicly available repositories can be queried to generate drug and disease signatures that can be compared to each other and integrated with newly generated experimental data **(c)**.

So far in computational drug discovery, drug response and disease phenotypes have been correlated with underlying pathological processes through ‘back-tracking’ approaches that can infer primary causes of transcriptional changes but require the integration of heterogeneous data sources and *a priori* known signalling and regulatory models [14–16]. Transcriptional profiles have also been used as a single data layer to dissect drug MoA through reverse-engineering techniques [17]. By contrast, recent studies suggest that purely data-driven approaches making use of gene expression data alone are well suited to identifying new drug repositioning opportunities. The leading idea is that comparing the expression profile of a cell before and after exposure can quantitatively assess the changes brought about by active compounds on the transcriptional programme. The corresponding signature of differential gene expression (SDE) can be considered as the summary of the compound's effect. Furthermore, a drug-induced SDE can then be compared with a disease-associated SDE similarly obtained through differential expression analysis of diseased versus healthy conditions. If they are sufficiently negatively correlated (i.e. the genes upregulated in the disease SDE are downregulated in the drug SDE and vice versa) then it is reasonable to hypothesise that the effect of the drug on transcription is opposite to the effect of the disease (Fig. 1a). As a consequence, the drug might be able to revert the disease SDE and hence the disease phenotype itself [18–20]. Alternatively, from a shared SDE it can be hypothesised that two drugs could share a therapeutic application, regardless of the similarity in their chemical structure and that they impinge on different intracellular targets or pathways [21–24] (Fig. 1b).

Despite the relative simplicity of these ideas, recent applications have shown that they could serve as the basis for identifying drug repositioning opportunities in different therapeutic areas to treat heterogeneous diseases from cancer [25,26] to Alzheimer's disease [24] and Crohn's disease [27].

In the following sections we examine how gene transcription profiles have been analysed in single case studies and we will describe several publicly available resources; finally we discuss challenges and future directions.

## Matching gene expression signatures to ‘connect’ phenotypes

Pioneering studies have shown that collections of gene sets (i.e. groups of genes sharing a common biological function, chromosomal location or regulation) can be used to interpret and extract biological insights from genome-wide expression profiles, by using parametric [28] or non-parametric statistical methods [29].

A genetic signature is defined by associating a gene set with a specific pattern of expression [30]. Gene expression profiling has been widely used as phenotype proxies [31], to build phenotype taxonomies [30,32], for systematic functional discovery [33] and for classification and/or cataloguing purposes [30,34]. Most importantly, gene expression signatures have been effective in recovering ‘connections’ between genes, drugs and diseases involving (or involved in) the same biological process, by combining a large collection of gene expression data following drug treatment with a pattern-matching method [35]. A seminal example of this is given by the Connectivity Map (cMap) [18,35], which is the first large public database of genome-wide gene expression profiles from five different human cancer cell lines treated with more than 1000 bioactive small molecules.

The aim of the cMap project was to generate a ‘map’ that can be searched for ‘connections’ between gene expression profiles associated with disease states and those following treatment with a large set of existing drugs. To query this map, the authors devised a pattern-matching tool based on Gene Set Enrichment Analysis (GSEA) [29] through which these connections can be inferred and statistically assessed.

**Figure 2:**
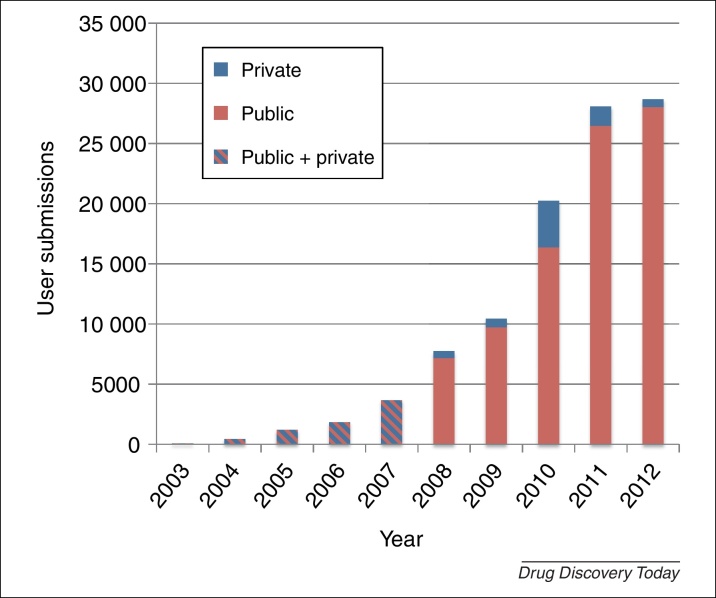
Rate of growth of ArrayExpress data in terms of experiments (i.e. user submission). This trend is set to increase further in the future, as new high-throughput sequencing-based transcriptomic applications result in the generation of huge amounts of data.

The effectiveness of this method for *in silico* drug discovery and drug repositioning has been demonstrated already by its very first applications [36,37], and it highlights the potential of gene transcription profiling to serve as the common language to link chemistry, biology and the clinic, by inferring genome-wide similarities or differences [35]. Numerous studies have been published using the cMap dataset and the cMap tool, with different aims (a comprehensive list is provided on the cMap website). This underscores the power of gene expression profiles and gene signatures in characterising biological states and acting as a surrogate phenotype, despite the difficulty in interpreting the meaning of predicted associations, let alone the precise part played by individual genes in these signatures [31]. Subsequent achievements have been to characterise the whole landscape of human gene expression [38], to establish large repositories of transcriptional data [39,40] and to make publically available a large amount of gene expression data that could be mined to compose drug and disease signatures (Fig. 2). Moreover, the robustness of these signatures has been shown across tissue types and experiments [41] and, during the past two years, the use of transcriptional data for drug repositioning has emerged as a useful and effective strategy [13,42], bringing about a new dawn for the vast quantities of DNA microarray data already in the public domain.

Although numerous approaches for *in silico* drug repositioning based on gene expression data have been published [19,20,22,24,25,27,36,37,43,44], all of them are methodologically similar and make use of the cMap SDEs as a reference database of drug responses in combination with signature-matching strategies. The majority of these methods can be subdivided into two main classes (features of which are summarised in Fig. 1). Methods in the first class aim to identify novel ‘drug–disease’ connection, whereas those in the second class aim to infer ‘drug–drug’ connections. In both cases gene expression profiles are used to summarise drug responses and disease states; and comparison between the two are based on the following simple but powerful assumptions:

(i) If an SDE summarising the response to a given approved drug is sufficiently negatively correlated to the SDE characterising a disease state, then that drug might be able to ‘revert’ the disease signature, hence the drug might be able to treat the disease phenotype. If already approved for other uses, the drug could be repositioned to treat that disease (Fig. 1a).
(ii) If two drugs elicit similar SDEs, even if acting on different intracellular targets, they could share a common MoA. In this case, the first drug could be repositioned to treat conditions for which the second drug has already been approved, or vice versa (Fig. 1b).

In the following section we will review case studies for methods in both classes.

### Reverting phenotype signatures to revert phenotypes

In this section we review methods based on the assumption that a drug that can revert a disease SDE might revert the disease phenotype itself. Building on this idea, several successful studies (methodologically similar to each other) identified new drugs for hepatocellular carcinoma [26], and were able to show the efficacy of vorinostat (currently used to treat cutaneous T-cell lymphoma) in treating gastric cancer [43] and also to predict several candidate therapeutics for cancer in a systematic manner [20,25].

Owing to their robust experimental validation and the methodological similarity with other cited works, here we concentrate on two representative case studies. The first is presented in a publication by Dudley *et al.* [27]. As a first step, the authors assembled an SDE for inflammatory bowel disease (IBD), which is a chronic inflammatory gastrointestinal disorder for which only few safe and effective drugs exist, from public gene expression data [40]. Then they mined the cMap dataset to identify drugs that had SDEs that opposed those of IBD. They then developed an algorithm to generate a ‘therapeutic score’ for each of the drugs in the cMap, applying a significance threshold value to determine when a drug SDE was opposite to the disease SDE. Among the top-ranked therapeutic predictions, the authors found not only the corticosteroid prednisolone (for which the efficacy in treating IBD has already been established and therefore defined as a positive control) but also, interestingly, a second candidate topiramate, an anticonvulsant drug approved for epilepsy, never linked before to IBD, which had a predicted therapeutic score higher than that of prednisolone. The authors used a trinitrobenzenesulfonic (TNBS) acid induced rodent model of IBD to validate their prediction *in vivo*. They showed that topiramate treatment improved damage in colon tissue which was one of the most severe symptoms of the induced phenotype. They therefore suggested that, given its safety profile, topiramate could indeed be repositioned to treat IBD in humans.

In a similar study, Kunkel *et al.* [45] generated SDEs of skeletal muscle atrophy, a condition currently lacking pharmacological therapy, by chronic fasting in human patients and mouse models. They used the resulting two SDEs to search the cMap database and both their queries returned ursolic acid as the only compound with an SDE opposite to that of the disease state. The authors went on to verify experimentally that ursolic acid reduced muscle atrophy and stimulated muscle hypertrophy in mice. They identified the MoA to be enhancement of skeletal muscle insulin/insulin-like growth factor-1 (IGF-1) signalling and inhibition of atrophy-associated skeletal muscle mRNA expression. Moreover, they observed additional effects on the characteristics of muscle following treatment with ursolic acid, including reductions in adiposity, fasting blood glucose, plasma cholesterol and certain triglycerides. These findings suggest a potential use of ursolic acid in muscle atrophy and other metabolic myopathies. With respect to the study by Dudley *et al.* [27], the methodological difference is that here the authors used a partial SDE composed only of genes with significant differential expression in skeletal muscle atrophy, rather than using a genome-wide SDE. Moreover, the authors used the cMap query tool for matching these partial SDEs to compounds rather than designing their own therapeutic score.

In the remainder of this section we describe how the signature reversion strategy has been used successfully to predict synergistic drug combinations when matching drug SDEs with SDEs characterising biological states other than diseases, thus highlighting the generality of such a method.

Motivated by the aim of reducing drug resistance of a cancer in a pharmacological way, Wei *et al.* successfully identified rapamycin as a modulator of glucocorticoid resistance in acute lymphoblastic leukaemia [46]. As in the previous cases, the first step was to identify an SDE representing a biological state to be ‘reverted’ by a drug. However, rather than using an SDE derived from a generic disease state, the authors derived a gene expression signature that differentiated acute lymphoblastic leukaemia samples sensitive to glucocorticoids from glucocorticoid-resistant samples, hence generating a drug resistance SDE rather than the SDE of a disease. Furthermore, the authors searched the cMap dataset for drugs with an SDE matching the signature they computed in an opposite way, identifying several potential active compounds. The top ranked drug in this list was rapamycin. Further analysis found that rapamycin elicits a sensitising action to glucocorticoids by acting on the antiapoptotic factor MCL1 (induced myeloid leukaemia cell differentiation protein).

In a similar study, Hassane *et al.* identified drugs that enhanced the antileukaemic effect of partenolide, a drug effective at reducing the survival and leukemogenic activity of primary human acute myeloid leukaemia stem cells [47]. However, partenolide induces cellular protective responses that reduce its cytotoxicity. As the starting point the authors selected a previously published SDE of response to partenolide. With this signature they queried the cMap database and they identified compounds acting along the phosphatidylinositol-3-kinase and mammalian target of rapamycin (mTOR) pathways among those eliciting an SDE similar to that of partenolide. Finally, they verified that treating acute myeloid leukaemia cells with a combination of partenolide and phosphatidylinositol-3-kinase/mTOR inhibitors was more effective than treating with partenolide alone at decreasing the viability of cells and tumour burden *in vitro* and in murine xenotransplantation models.

Taken together, these studies clearly show the potential of ‘signature reversion’ in identifying new uses for existing drugs as well as to predict novel chemosensitising effect and synergistic drug combinations.

### Mining similarity of transcriptional responses to drugs to identify a shared MoA

In contrast to the examples in the previous section, here we review approaches based on the assumption that if two drugs elicit similar transcriptional responses then they could share a MoA and hence could be applied to the same pathological condition.

Inferring drug target binding by comparing the molecular similarity of sets of candidate drug compounds has been a traditional approach in drug discovery. This is ligand-based drug design and has been most often applied when structural information regarding the target proteins and their binding sites are absent. Candidate drug compounds known to inhibit the same target protein can be compared using their chemophoric (interaction) patterns. However, only when the 3D geometries of their interaction patterns match can a pharmacophore (the complementary set of binding interactions) be inferred representing the possible shared binding site of the target protein. Comparisons of interaction geometries can be seen in the literature reporting QSAR and comparative molecular field analysis (CoMFA) studies of two or more drug compounds that share a common target or equivalent binding sites in homologous proteins [48]. Conversely, a comparison of binding sites known to be targeted by one set of inhibitors and drugs can be used to infer equivalent binding sites in new targets. Consequentially, the target of a new drug can, in principle, be deduced by looking at the targets of the drugs most similar to it.

This ‘guilt-by-association’ principle has been successfully applied to identify new targets for existing drugs [8], by defining the corresponding set of ligands for a large number of known targets and then computing chemical similarities between drugs and ligand sets. In addition to structural similarity, the same principle has been applied to exploit other kinds of drug similarity in MoA discovery and repositioning in structural bioinformatics [9] where proteins with similar binding sites are targeted by the same drug; text mining [10], where two drugs sharing a semantic concept are assumed to share a therapeutic application; recently, ‘modulatory profiling’ [23], measuring changes in efficacy of lethal compounds when used in combination with a second cell-death-modulating agent (here drugs with similar modulatory profiles could have the same MoA); finally, as mentioned above, gene expression data, where two drugs elicit a similar SDE and could have a common MoA even if they act on different intracellular targets [22].

Based on the premise of shared genome-wide molecular activity, Iorio *et al.* [22] systematically compared all the cMap drugs in a pair-wise fashion, rather than searching for drugs eliciting an SDE similar or opposite to an input signature. By doing this they identified a large number of drug–drug ‘associations’ based on the extent of similarity between the corresponding SDEs. By making use of a novel similarity score, they constructed a network representation in which each node is a drug and each edge (connection) indicates a significant similarity between the SDEs of the connected nodes. They divided the drug network into groups of densely interconnected nodes termed ‘communities’, containing drugs eliciting similar SDEs. Communities were strikingly populated by drugs with similar known MoAs or sharing a therapeutic application.

The authors demonstrated the power of their method to identify the MoA of novel drugs by analysing their neighbouring communities once they were integrated in the network. In a similar way, they showed how the drug network could be used to infer new applications for already existing drugs by searching subnetworks surrounding a drug with a desired MoA for other compounds never linked before to that MoA. By doing this, they were able to predict and experimentally verify that fasudil, a safe Rho-kinase inhibitor approved in Japan to reverse blood vessel obstructions after ischemic stroke, can enhance cellular autophagy [21], a metabolic process implicated in several neurodegenerative disorders.

A related method was proposed by Hu and Agarwal [24], who inferred a drug–disease network in which two nodes were connected by an edge if the corresponding SDEs were significantly similar (in the case of drug–drug connections) or significantly negatively correlated (in the case of drug–disease connections). To achieve this, the authors integrated the SDE of the cMap drugs with a large number of disease SDEs assembled by mining the Gene Expression Omnibus (GEO) repository [40]. Connections representing anticorrelations were predictive of new indications for existing drugs, such as the potential use of some antimalarial drugs for Crohn's disease, and the possible repositioning of several existing drugs as therapeutic options for Huntington's disease. This approach can be seen as a precursor hybrid method, mixing together the two types of approaches of disease signature reversion and guilt-by-association. Moreover, the authors hypothesise that drug side effects could be predicted by the analysis of similarity between drug and disease SDEs.

In conclusion, the results presented show how large collections of gene expression data following drug treatment could be exploited through a guilt-by-association approach with the aim of identifying drug repositioning opportunities.

## Resources for computational expression-based drug repositioning

Several resources support computational drug repositioning based on transcriptional data and the functional characterisation of gene sets and signatures. Some freely available tools and database are listed in Table 1.

ArrayExpress [39], GEO [40] and the cMap [18,35] are large public repositories of gene expression data from where disease and drug-response signatures can be assembled. DAVID [49], MsigDB [29] and GeneSigDB [50] are useful tools for functionally characterising large gene lists by using pre-defined functional terms, or pre-defined gene signatures representing different biological entities and processes from public repositories. These signatures can also be used to characterise with regard to function large sets of differentially expressed genes from microarray studies through non-parametric statistical methods that can also provide complementary information, such as the GSEA tool [29] or Expression Analysis System Explorer (EASE) and regulatory motif analysis [51,52].

The cMap query tool has two extensions: sscMap [53,54] and the MoA by network analysis (MANTRA) tool [22]. sscMAP is a free-to-download java implementation of the cMap algorithm bundled with the reference dataset, enabling the integration of user-defined data. MANTRA makes use of a post-processed version of the cMap dataset, where compounds are catalogued into a drug similarity network. In this network two drugs are connected if they elicit a similar transcriptional response in human cell lines. With MANTRA users can integrate a drug under investigation into the network and deduct its MoA by analysing the surrounding subnetwork. Moreover, it is possible to identify drug repositioning opportunities by searching the neighbourhood of a ‘seed’ compound with a desired MoA for ‘safe’ compounds never linked before to that MoA.

Several tools are freely available for mining gene expression data repositories based on similarity with an input signature in a similar manner to cMap. ProfileChaser [55] searches microarray repositories based on genome-wide patterns of differential expression, and MARQ [56] mines GEO for experiments that generate a differential expression profile that is similar or anti-correlated to an input gene expression signature. Finally, DvD is a recently developed tool providing a pipeline for the comparison of drug and disease gene expression profiles from public microarray repositories.

## Challenges of signature-matching methods

A potential major problem affecting the methods described here is the challenge of integrating independent microarray studies. Microarrays do not measure gene expression in absolute units. As a consequence, an improper handling of multiple gene expression profiles obtained in different experimental settings would capture similarities in these settings rather than in the represented biological states (a phenomenon known as the ‘batch effect’ [57]). By contrast, cells in different pathological conditions or with different genomic backgrounds respond very differently to the same drug treatment. Consequently, classic microarray analysis approaches might not produce optimal results, because they tend to discriminate gene expression profiles on the basis of the experimental settings in which they have been produced rather than on the basis of the stimuli they are responding to (for example a drug treatment).

In most of the methods we describe in this review, these problems are partially addressed by making use of non-parametric statistics [29], genome-wide ranked lists of genes [18] and ‘consensual responses’ to drugs [22] rather than classic similarity metrics applied to individual profiles of expression values or fold-change-derived significance scores. However, a potential drawback of these techniques is that they might dilute cell-specific ‘gene expression signals’ by pooling together the transcriptional response to the same drug but from different experimental settings (i.e. different cell lines, dosages or observation times). These problems have been tackled by designing *ad hoc* similarity scores [58] and genome-wide metrics [44,59,60].

RNA-seq technology might overcome many of these limitations, because it can detect amounts of RNA over a wider dynamic range. In the long run, RNA-seq could replace microarrays for SDE analysis; meanwhile use of microarray data remains attractive, being not only a simpler and more cost-effective technology but also one with a vast collection of already publicly available data.

## Concluding remarks

We have reviewed approaches using microarray data to assist in the elucidation of compound MoA with the specific goal of identifying new potential applications for existing drugs. A significant number of published results show that microarray technology provides a unique opportunity to identify repositionable drugs by exploiting the vast amount of existing publicly available data where the potential has not yet been fully capitalised.

The methods we described do not consider mechanistic aspects, but simply use transcriptional signatures as readouts from the ‘black-box’ of cellular mechanisms. Therefore, they cannot provide any information about cell signalling pathways where a deregulation can result in an observed expression signature.

It could be argued that as long as the drug works the mechanism is a secondary consideration. But at the same time, it is reasonable to expect that additional insight into a MoA for a given drug can be obtained by integrating expression data with knowledge of (and ideally data from) the systems in which the drugs operate, known regulatory relationships between genes and signalling pathway maps.

The challenge for the future will be to take current analyses to a higher level, integrating signatures and mechanistic insights inferred by other recently developed approaches. This will require repositories of comprehensive gene expression data for disease states and compound effects, and integration with prior knowledge of cellular networks on which drugs operate, and further development of computational methods to translate this data into effective medicines.

So far, recent results encouragingly illustrate that computational approaches using public gene expression microarray data can be successfully employed to infer new potential drug therapies. We argue that this can (and probably will) be further exploited in the near future.

## Figures and Tables

**Table 1:** Publicly available resources to derive, compare and functionally characterise gene expression signatures

**Resource**	**Short description**	**Minable for partial and genome-wide signatures of drug responses and disease states**	**Tool for signature matching and classification of microarray data**	**Functional characterisation of gene sets/signatures**	**Oriented to drug-discovery and repositioning**	**Website**
**ArrayExpress**	Public repositories of gene expression data	✓				http://www.ebi.ac.uk/arrayexpress/
**Gene Expression Omnibus – GEO**				http://www.ncbi.nlm.nih.gov/geo/
**Database for Annotation, Visualization and Integrated Discovery – DAVID**	Functional annotation tools to associate biological meaning to list of genes through analysis of over-represented terms			✓		http://david.abcc.ncifcrf.gov/
**Gene Expression Atlas**	Subset of ArrayExpress archive, servicing queries for condition-specific gene expression patterns	✓	✓			http://www.ebi.ac.uk/gxa/
**Molecular Signature Database – MsigDB**	Collections of annotated gene signatures from different sources	✓		✓		http://www.broadinstitute.org/gsea/msigdb
**Gene Signature Database – GeneSigDB**			http://compbio.dfci.harvard.edu/genesigdb/
**Gene Set Enrichment Analysis – GSEA**	Tool able to determine if an *a priori* defined gene signature shows statistically significant, concordant differences between two biological states		✓	✓		http://www.broadinstitute.org/gsea
**ProfileChaser**	Tools to search the GEO repository for experiments whose differential expression looks similar or opposite to a gene expression signature or a query experiment	✓	✓			http://profilechaser.stanford.edu/
**MicroArray Rank Query – MarQ**			http://marq.dacya.ucm.es/
**Connectivity Map – cMap**	Large collection of gene expression data following drug treatment that can be queried with an integrated pattern-matching tool, based on GSEA, to find drugs eliciting a response similar or opposite to a given gene signature	✓	✓		✓	http://www.broadinstitute.org/cmap/
**Statistically significant connections’ map – sscMap**	Java implementation of the cMap tool bundled with the corresponding dataset and making it extendable with adding custom collections of reference profiles		✓	✓		http://purl.oclc.org/NET/sscMap
**Mode of Action by NeTwoRk Analysis – MANTRA**	Tool for the analysis of the mode of action of novel drugs and the identification of drug repositioning opportunities, based on network theory and GSEA and making use of a post-processed version of the cMap database	✓	✓		✓	http://mantra.tigem.it
**Drug versus Disease – DvD**	Computational pipeline for comparing disease and drug-response gene expression signatures from publicly available resources	✓	✓		✓	www.ebi.ac.uk/saezrodriguez/dvd
